# Expression of Wnt/*β*-Catenin Signaling Pathway and Its Regulatory Role in Type I Collagen with TGF-*β*1 in Scleral Fibroblasts from an Experimentally Induced Myopia Guinea Pig Model

**DOI:** 10.1155/2016/5126560

**Published:** 2016-05-10

**Authors:** Min Li, Ying Yuan, Qingzhong Chen, Rao Me, Qing Gu, Yunjie Yu, Minjie Sheng, Bilian Ke

**Affiliations:** ^1^Department of Ophthalmology, Shanghai First People's Hospital, Shanghai Jiao Tong University School of Medicine, Shanghai, China; ^2^Department of Ophthalmology, Yangpu Hospital, Tongji University, Shanghai, China

## Abstract

*Background*. To investigate Wnt/*β*-catenin signaling pathway expression and its regulation of type I collagen by TGF-*β*1 in scleral fibroblasts from form-deprivation myopia (FDM) guinea pig model.* Methods*. Wnt isoforms were examined using genome microarrays. Scleral fibroblasts from FDM group and self-control (SC) group were cultured. Wnt isoforms, *β*-catenin, TGF-*β*1, and type I collagen expression levels were examined in the two groups with or without DKK-1 or TGF-*β*1 neutralizing antibody.* Results*. For genome microarrays, the expression of Wnt3 in FDM group was significantly greater as confirmed in retinal and scleral tissue. The expression of Wnt3 and *β*-catenin significantly increased in FDM group and decreased significantly with DKK-1. TGF-*β*1 expression level decreased significantly in FDM group and increased significantly with DKK-1. Along with morphological misalignment inside and outside cells, the amount of type I collagen decreased in FDM group. Furthermore, type I collagen increased and became regular in DKK-1 intervention group, whereas it decreased and rearranged more disorder in TGF-*β*1 neutralizing antibody intervention group.* Conclusions*. The activation of Wnt3/*β*-catenin signaling pathway was demonstrated in primary scleral fibroblasts in FDM. This pathway further reduced the expression of type I collagen by TGF-*β*1, which ultimately played a role in scleral remodeling during myopia development.

## 1. Introduction

Myopia has emerged as a major global public health issue, and it can increase the risk of irreversible visual impairment that would be a serious threat to human health and quality of life [1]. Myopia development is closely related to the continuous expansion and thinning of the sclera. Previous studies have indicated that biochemical and biomechanical properties of the sclera determine the shape and size of eye globe and therefore play a major role in influencing the refractive state of the eye [2, 3]. Sclera tissue is composed of extracellular matrix (ECM) and fibroblasts that secrete matrix [4]. ECM from scleral fibroblasts is involved in scleral remodeling process [5], and it mainly consists of type I collagen [2, 6]. Therefore, ECM, especially type I collagen, plays an important role in maintaining the basic form to prevent pathologic myopia [7]. Previous studies have reported the common regulatory sites in scleral ECM in myopia. However, the mechanism for sclera remodeling still needs further investigation.

The Wnt/*β*-catenin pathway is currently recognized as particularly important in embryogenesis and tissue regeneration [8]. Research has shown that Wnt/*β*-catenin pathway has functional links to the retina, choroid, and sclera through multiple cytokines, which play an important role in maintaining eye development after birth [9–12]. The crosstalk between Wnt/*β*-catenin pathway and other three pathways, BMPs, Hedgehogs, and FGFs, played key roles during development of body tissues and organs [8, 13–15]. These other three pathways have been identified in myopia development [16–20]. A previous study found that the amounts of Wnt2b and *β*-catenin in mRNA and protein from retina were significantly greater in FDM eyes than in control eyes [21]. However, to our knowledge, no study has fully determined the mechanism of Wnt/*β*-catenin pathway in regulating the process of myopia development. Moreover, Wnt/*β*-catenin signaling pathway is associated with several conditions related to ECM, such as organ fibrosis [22, 23], scar tissue formation [24], and diabetic retinopathy fibrosis [25]. This signaling pathway is also involved in the expression of types I, II, and III collagen [22, 26]. Furthermore, regulatory factors in Wnt/*β*-catenin pathway for regulating the sclera ECM need to be discussed in order to suggest potential strategies for therapeutic purposes in myopia.

Transforming growth factor- (TGF-) *β*1 is a key regulator in fibrosis diseases and can induce the expression of components of ECM, especially type I collagen [27]. The Wnt/*β*-catenin signaling pathway could affect the expression of TGF-*β*1 in fetal and postnatal fibroblasts [28]. Moreover, the interaction between TGF-*β*1 and Wnt/*β*-catenin signaling pathway has an important role in regulating the expression of collagen [29, 30]. TGF-*β*1 is also reported to be involved in the regulation of scleral remodeling [31]. However, the novel link between Wnt/*β*-catenin signaling pathway and TGF-*β*1 in scleral remodeling related to myopia remains unclear.

Our study aims to investigate the regulatory mechanism of Wnt/*β*-catenin signaling pathway in the expression of collagen by TGF-*β*1 in scleral fibroblasts from FDM to provide evidence for seeking new intervention targets in the pathogenesis of scleral remodeling in myopia.

## 2. Materials and Methods

### 2.1. Animals

Two-week-old pigmented guinea pigs were obtained from the Shanghai Laboratory Animal Center (Shanghai, China). All animals in our study were treated according to the ARVO Statement for the Use of Animals in Ophthalmic and Vision Research and were approved by the Ethics Committee of Shanghai General Hospital, Shanghai Jiao Tong University School of Medicine, Shanghai, China (Permit Number: 2009-0086). All animals were clinically examined to confirm corneal transparency of each eye and lack of injuries or infections in the eyes. Guinea pigs were reared on a cycle of 12 h illumination and 12 h darkness daily during the experimental period with freely available food and water.

### 2.2. Development of FDM

Thirty pigmented guinea pigs were randomly divided into two groups: the FDM group and the normal control (NC) group. In the FDM group, animals wore a translucent diffuser on one eye as previously reported [32, 33]. The fellow eye of the same guinea pig was untreated as the self-control (SC) group. The diffusers were checked every day to ensure that they were in place. Animals in the NC group were raised in the same conditions, with both eyes untreated. In the induction periods of 1, 2, 3, and 4 weeks, refractive errors were examined using cycloplegic streak retinoscopy. The axis lengths were measured by A-ultrasonic scanning (KN-1800, KangNing, China). All examinations were performed by two doctors independently and were then repeated three times to obtain the reported average value.

### 2.3. Whole Genome Microarrays

After the successful establishment of the FDM guinea pig model, the retinal tissues in both the FDM group and the SC group were retrieved using microscissors. RNA was isolated using Trizol (Invitrogen, USA) according to the manufacturer's instructions. Whole genome microarrays in both the FDM group and SC group were used by the Gene Expression Microarray (GPL18607, Agilent, USA) according to the manufacturer's instructions. The extracted data were analyzed using the Agilent Feature Extraction Software.

### 2.4. Primary Culture of Scleral Fibroblasts

After myopia induction, eyes in both the FDM group and the SC group were immediately removed from each guinea pig under sterile conditions and rinsed with saline that contains gentamicin and phosphate-buffered saline (PBS) containing 10% penicillin and streptomycin three times. The scleral tissues were collected using microscissors, cut into 1 mm × 1 mm pieces, and carefully placed into separate flasks in DMEM (High Glucose) (Gibco, USA) containing 10% fetal bovine serum (FBS) (Gibco, USA), 100 U/mL penicillin, and 100 *µ*g/mL streptomycin (Gibco, USA). Cells of 80% confluence were passaged by 0.25% trypsin-EDTA (Gibco, USA). The cells primary cultured were identified with vimentin (Boster Biological Technology, China) and keratin antibodies (Santa, USA). Cells of 80% confluence were washed and cultured with DMEM (High Glucose) without FBS for 24 h and then incubated with the basal medium plus 100 ng/mL DKK-1 (Wnt/*β*-catenin pathway antagonist) (R&D, USA) or 1 *µ*g/mL TGF-*β*1 neutralizing antibodies (Abcam, UK) for 48 h to extract proteins or collect the supernatant.

### 2.5. Immunocytochemistry

Scleral fibroblasts grown on coverslips were immersed in 4% paraformaldehyde for 30 min. The coverslips were washed for 5 min in PBS three times. Subsequently, 0.1% Triton X-100 was added, and the cells were incubated at room temperature for 20 min. The cells were washed three times with PBS, for 5 min each time. They were then incubated for 15 min with 3% H_2_O_2_ and washed three times (5 min each time) using PBS. The cells were blocked in 5% BSA for another 20 min. To stain the vimentin and keratin, the cells were incubated overnight with primary antibodies in PBS at 1 : 100 and 1 : 50 dilutions at 4°C. After being washed three times with PBS for 5 min each time, the samples were incubated for 20 min with secondary antibodies at 37°C and then washed three times with PBS for 2 min each time. The cells were then incubated for 30 min with streptavidin-biotin complex at 37°C and washed three more times with PBS for 5 min each time. After staining the cells with diaminobenzidine for approximately 3 min, distilled water was added to stop the reaction. PBS was used instead of the primary antibody for the blank control.

### 2.6. Western Blot

Cells or tissues were sonicated in RIPA buffer containing protease inhibitors. The supernatants were collected after centrifugation. Proteins of an equal concentration were separated on 10% SDS-PAGE and transferred to a PVDF transfer membrane (Millipore Corporation, Temecula, CA). The membranes were blocked in TBS containing 0.1% Tween-20 and 5% nonfat dry milk for 90 min, followed by an overnight incubation at 4°C with Wnt3 antibody (Proteintech, USA) at a 1 : 1000 dilution, *β*-catenin antibody (R&D, USA), and type I collagen antibody (Proteintech, USA) at a 1 : 250 dilution. After a rinse in TBST, the membranes were incubated for 1 h with a horseradish peroxidase-conjugated secondary antibody against rabbit or mouse IgG (Dako, Denmark) in a 1 : 5000 dilution. Then, they were rinsed with TBST followed by SuperSignal West Pico Chemiluminescent Substrates (Pierce, USA) for detecting the blots. The densities of the bands were analyzed by the Gel-Pro Analyzer. The expression of *β*-actin was used as an internal control.

### 2.7. Enzyme-Linked Immunosorbent Assay (ELISA)

Cell supernatant concentrations were measured by a TGF-*β*1 ELISA kit (R&D, US). The optical density (OD) was measured at 450 nm on a microtiter plate reader. All measurements were performed twice, and the mean values were computed.

### 2.8. Immunofluorescence

Scleral fibroblasts grown on coverslips were immersed in 4% paraformaldehyde for 30 min. The coverslips were washed three times for 5 min each time in PBS. The cells were incubated overnight with type I collagen antibody (Abcam, UK) at a 1 : 500 dilution at 4°C. After being washed three times with PBS for 5 min each time, the samples were then incubated for 1 h with secondary antibodies at a 1 : 100 dilution at room temperature and then washed three times with PBS for 2 min each time. The cells were stained with PI for approximately 5 min and then observed with immunofluorescence microscopy.

### 2.9. Data Analyses

All results were expressed as the mean ± standard error (SE). Comparisons between the FDM and the SC groups were made using an independent *t*-test, which was also used to compare the right eye and the left eye in the NC group. One-way ANOVA (analysis of variance) was used to make comparisons among the groups after intervention. *P* < 0.05 was considered statistically significant. All statistical routines were used as implemented in SPSS version 19.0.

## 3. Result

### 3.1. Wnt Isoforms in Experimentally Induced Myopia Guinea Pig Model

**Figure 1:**
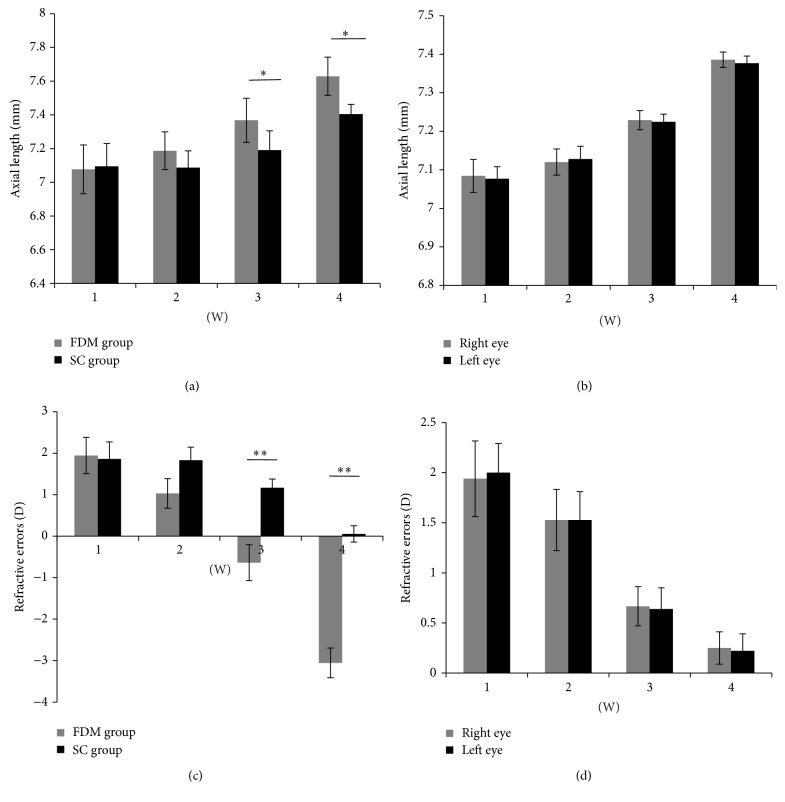
Axial length and refractive errors in FDM group, SC group, and NC group at 1 week, 2 weeks, 3 weeks, and 4 weeks. (a) Axial length compared FDM group with SC group. (b) Axial length compared right eye with left eye in NC group. (c) Refractive errors compared FDM group with SC group. (d) Refractive errors compared right eye with left eye in NC group. ^*∗*^ *P* < 0.05, ^*∗∗*^ *P* < 0.01.

During the first and the second weeks of modeling, no significant differences in axial length and refraction errors were found between the FDM group and the SC group (*P* > 0.05). In the third week, axial length in the FDM group increased significantly (*P* < 0.05) and refraction errors in the FDM group decreased significantly (*P* < 0.01) compared with those in SC group. In the fourth week, axial length in the FDM group was still longer than that in the SC group (*P* < 0.05), and refraction errors were still reduced (*P* < 0.01) (Table 1, Figure 1). In the NC group, no significant difference was observed in axial length and refractive errors of the two eyes of each guinea pig for all four weeks (*P* > 0.05) (Table 2, Figure 1).

**Figure 2:**
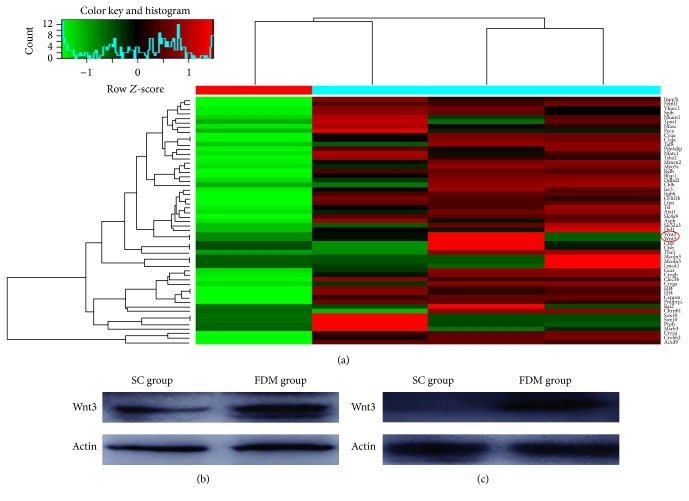
Expression of Wnt isoforms in form-deprivation myopia guinea pig model. (a) The result of whole genome microarrays. (b) Western blot for the expression of Wnt isoforms in retinal tissue. (c) Western blot for the expression of Wnt isoforms in scleral tissue.

The genome microarray result showed that the expression of Wnt3 was 2.13 times greater in the FDM group than in the SC group. Wnt3 also increased in the retina and scleral tissue in the FDM group observed by Western blot, consistent with the result of genome microarray (Figure 2).

### 3.2. Expression of Wnt3/*β*-Catenin Signal Pathway in Scleral Fibroblasts from Experimentally Induced Myopia Guinea Pig Model

Starting on the third day, scleral fibroblasts grew out of scleral tissue pieces, which were stellate-shaped, and became spindle-shaped after six days of being cultured. On the ninth day, the tissues were removed, and the cells were fused 80%–90%. On the fifth day at the second passage, cell fusion was approximately 80%–90%.

**Figure 3:**
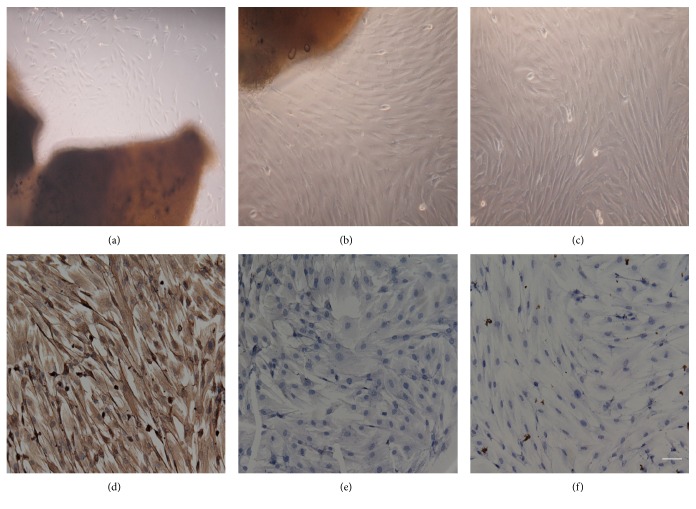
Primary culture and identification of scleral fibroblasts in guinea pig. (a) At the third day, a small amount of fibroblasts climbed out from the scleral tissue. (b) At the sixth days, there were about 60% of fibroblasts. (c) At the fifth days at the second passage, cell fusion was about 80%–90%. (d) Cultured scleral fibroblasts were stained positive for vimentin. (e) In blank control group, the staining in cultured scleral fibroblasts was negative. (f) Cultured scleral fibroblasts were stained negative for keratin (original *∗* 200, bar = 50 *µ*m).

Through immunocytochemical staining, scleral cells cultured showed brownish yellow, which means positive staining for vimentin and negative staining for keratin and PBS. These results confirmed that the cells cultured were fibroblasts [27] (Figure 3).

**Figure 4:**
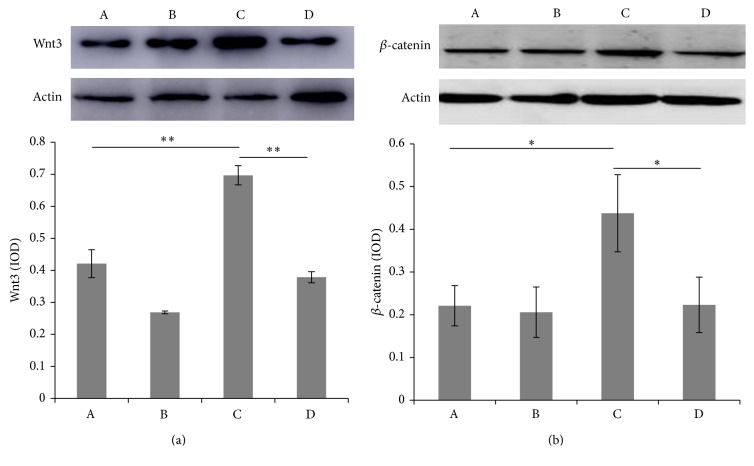
Western blot for the expression of Wnt3 and *β*-catenin in guinea pig scleral fibroblasts. (a) Western blot for the expression of Wnt3. (b) Western blot for the expression of *β*-catenin. (A) SC group, (B) SC + DKK-1, (C) FDM, and (D) FDM + DKK-1. ^*∗∗*^ *P* < 0.01, ^*∗*^ *P* < 0.05.

As observed by Western blot, the expressions of Wnt3 and *β*-catenin significantly increased in the FDM group compared with SC group (*P* < 0.05). The expressions significantly decreased in the FDM group with DKK-1 added (*P* < 0.05) (Figure 4).

### 3.3. Regulation of the Wnt3/*β*-Catenin Signal Pathway in the Expression of TGF-*β*1 and Type I Collagen

**Figure 5:**
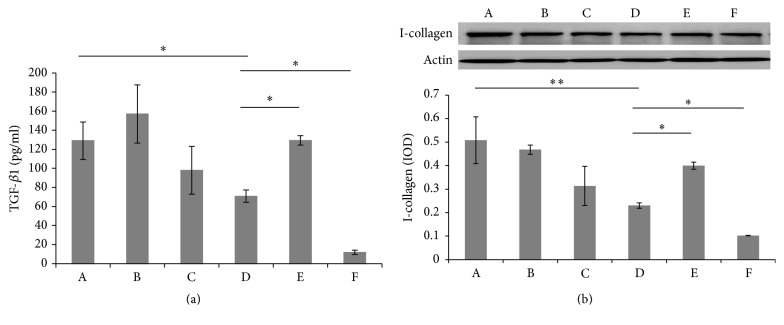
The expression of TGF-*β*1 and type I collagen in guinea pig scleral fibroblasts. (a) ELISA for the expression of TGF-*β*1 in supernatant of guinea pig scleral fibroblasts. (b) Western blot for the expression of type I collagen in scleral fibroblasts. (A) SC, (B) SC + DKK-1, (C) SC + TGF-*β*1 neutralizing antibody, (D) FDM, and (E) FDM + DKK-1. (F) FDM + TGF-*β*1 neutralizing antibody (^*∗*^ *P* < 0.05, ^*∗∗*^ *P* < 0.01).

As observed by ELISA, the expression of TGF-*β*1 in cell supernatant decreased more significantly in the FDM group than in the SC group (*P* < 0.05). However, its expression increased significantly with DKK-1 (*P* < 0.05) but decreased further with the TGF-*β*1 neutralizing antibody in the FDM group (*P* < 0.05) (Figure 5(a)).

As observed in Western blot, type I collagen in the FDM group decreased significantly compared with the SC group (*P* < 0.01). After adding DKK-1, type I collagen in the FDM group significantly increased (*P* < 0.05). After adding the TGF-*β*1 neutralizing antibody, the expression of type I collagen further reduced (*P* < 0.05) (Figure 5(b)).

**Figure 6:**
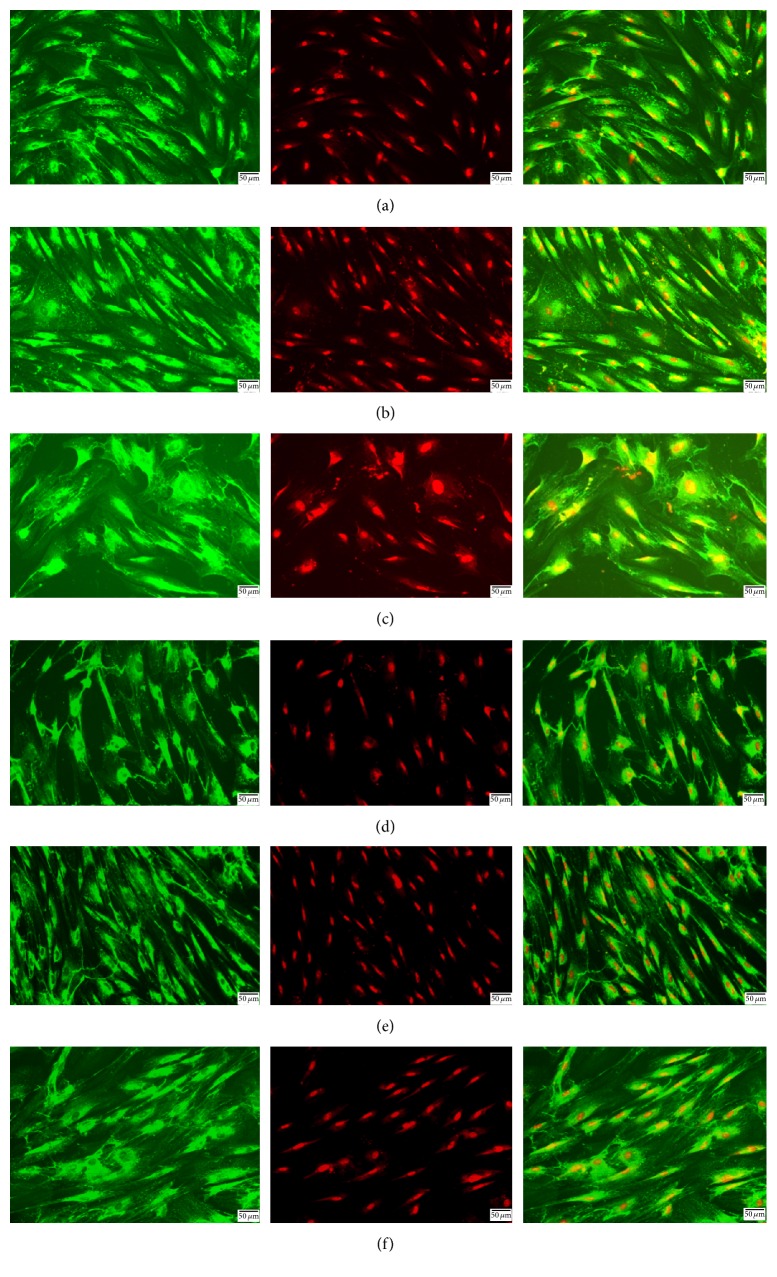
Immunofluorescence for the expression of type I collagen in guinea pig scleral fibroblasts. (a) SC group. (b) SC group added with DKK-1. (c) SC group added with TGF-*β*1 neutralizing antibody. (d) FDM group. (e) FDM group added with DKK-1. (f) FDM group added with TGF-*β*1 neutralizing antibody (green for type I collagen, red for PI) (original *∗* 200, bar = 50 *µ*m).

As observed by immunofluorescence, the arrangement of type I collagen was more disordered in the FDM group than in the SC group, and its expression in the FDM group also decreased. After adding DKK-1, the expression of collagen increased, and the arrangement was more regular. However, the arrangement of type I collagen was still disordered with the TGF-*β*1 neutralizing antibody (Figure 6).

## 4. Discussion

Myopia is a common ocular condition, characterized by the excessive elongation of the ocular globe [34]. Experimental evidence indicates that excessive ocular elongation in myopia is associated with scleral remodeling, which is considered as an important breakthrough in the etiology of experimental myopia [2, 3]. Furthermore, the regulation mechanisms of scleral remodeling still need to be investigated. Wnt/*β*-catenin pathway plays a critical role in the development of body tissues and organs. This pathway has a functional relationship with the cytokines in the retina, choroid, and sclera, which play an important role in maintaining eye development [9–12]. Although the early theory predicted that Wnt/*β*-catenin pathway may be involved in the process of FDM [21], the regulatory mechanism of Wnt/*β*-catenin pathway in experimental myopia still remains unclear. Moreover, the downstream sites of Wnt/*β*-catenin pathway, which ultimately influences the scleral collagen expression, need to be further investigated.

In this study, Wnt/*β*-catenin signaling pathway was mainly induced by Wnt3 to regulate experimentally induced myopia. Wnt3 is one of canonical Wnts for activating Wnt/*β*-catenin signaling pathway [35]. Research has shown that Wnt3 is a biomarker capable of predicting embryonic stem cell differentiation and tissue development [36, 37]. Wnt3 was detected in the neural retina, lens, and eyelid [38, 39]. Our study firstly demonstrated that Wnt3 was expressed in scleral fibroblasts from FDM, which was considered to mediate canonical Wnt/*β*-catenin signaling pathway in myopia.

TGF-*β* is a key factor in regulating ECM expression [27], and TGF-*β*1 has a regulatory effect on scleral remodeling related to myopia [31]. Moreover, Wnt and TGF-*β* are involved in a balanced network to regulate the optic vesicle development, neuron differentiation, and retinal stem cell survival [40]. Wnt/*β*-catenin signaling pathway can also affect the expression of TGF-*β*1 in fetal and postnatal fibroblasts [28]. Our study found that TGF-*β*1 produced in the FDM group was significantly lower than that in the control group, but it significantly increased after the addition of DKK-1, Wnt/*β*-catenin pathway antagonist. Therefore, Wnt3/*β*-catenin signaling pathway, as the upstream regulatory pathway, regulated the expression of TGF-*β*1 in scleral fibroblasts in experimental myopia.

Scleral remodeling is a dynamic process that involves continual synthesis and degradation of ECM produced by scleral fibroblasts. Type I collagen-dominated ECM is important in scleral remodeling in myopia [5]. In addition, TGF-*β*1 mediates the communication among cells to make a regulatory effect in scleral remodeling related to myopia [7, 31]. In some tissues, TGF-*β*1 interacts with Wnt/*β*-catenin signaling pathway, which has a certain regulatory effect on the expression of collagen in human airway and skin cells [29, 30]. However, no report has been conducted on whether or not Wnt/*β*-catenin signaling pathway is involved in the regulation of type I collagen expression with TGF-*β*1 in the development of FDM.

First, our study found that, along with the morphological misalignment inside and outside cells, type I collagen decreased significantly in the FDM group. After the addition of DKK-1, the expression of type I collagen increased, and the intracellular and intercellular collagen arrangements became orderly. Therefore, the Wnt3/*β*-catenin signaling pathway was presumed to regulate the pathological scleral remodeling process by affecting the expression of type I collagen. Second, in the FDM group treated with the TGF-*β*1 neutralizing antibody, the expression of type I collagen was further reduced, and the arrangement of collagen was more cluttered. We considered that TGF-*β*1, as the downstream factor of the Wnt3/*β*-catenin signaling pathway, could be involved in transmembrane bridges to mediate the internal and external communication between cells and ECM for regulating scleral remodeling.

In summary, our study found that Wnt3/*β*-catenin signaling pathway was activated during the development of FDM and that it could reduce the expression of TGF-*β*1 to mediate type I collagen-dominated ECM in the sclera. Furthermore, our results on the potential relationship between FDM and Wnt3/*β*-catenin signaling pathway clearly suggest a potential target for preventing and treating scleral remodeling in myopia.

## Figures and Tables

**Table 1:** The refractive errors and axial length in FDM group and SC group.

Induction time (weeks)	Number	Refractive errors (D)			Axial length (mm)		
		FDM	SC	*P*	FDM	SC	*P*
1	20	1.94 ± 1.31	1.86 ± 1.24	>0.05	7.08 ± 0.15	7.09 ± 0.14	>0.05
2	20	1.03 ± 1.07	1.83 ± 0.94	>0.05	7.19 ± 0.11	7.09 ± 0.10	>0.05
3	18	−0.64 ± 1.30	1.17 ± 0.64	<0.01	7.38 ± 0.13	7.16 ± 0.11	<0.05
4	18	−3.06 ± 1.08	0.06 ± 0.60	0.01	7.63 ± 0.11	7.40 ± 0.06	0.01

FDM: form-deprivation myopia. SC: self-control.

**Table 2:** The refractive errors and axial length in NC group.

Induction time (weeks)	Number	Refractive errors (D)			Axial length (mm)		
		Right eye	Left eye	*P*	Right eye	Left eye	*P*
1	10	1.94 ± 1.13	2.00 ± 0.88	>0.05	7.08 ± 0.13	7.08 ± 0.09	>0.05
2	10	1.53 ± 0.91	1.53 ± 0.85	>0.05	7.12 ± 0.10	7.13 ± 0.10	>0.05
3	9	0.67 ± 0.64	0.64 ± 0.59	>0.05	7.23 ± 0.07	7.22 ± 0.06	>0.05
4	9	0.25 ± 0.48	0.22 ± 0.51	>0.05	7.39 ± 0.06	7.38 ± 0.06	>0.05

NC: normal control.
